# Editorial: Public health dentistry and oral infectious disease dynamics, diagnosis and management

**DOI:** 10.3389/fpubh.2023.1289584

**Published:** 2023-10-09

**Authors:** Dominic Augustine, S. V. Sowmya, H. N. Yukta, Shankargouda Patil

**Affiliations:** ^1^Department of Oral & Maxillofacial Pathology and Oral Microbiology, Faculty of Dental Sciences, M. S. Ramaiah University of Applied Sciences, Bengaluru, India; ^2^College of Dental Medicine, Roseman University of Health Sciences, South Jordan, UT, United States

**Keywords:** oral infectious disease, oral disease dynamics, oral disease, oral disease management, public health dentistry

The Research Topic “*Public health dentistry and oral infectious disease dynamics, diagnosis, and management*” covers critical oral disease etiological factors and treatment outcomes that have a broad impact on public health and society as a whole. This issue does not only offer insights on the complexities of infectious diseases, but also prioritizes the importance of improving community wellbeing and public health outcomes.

Oral infectious disease dynamics constitute the involvement of interaction with oral microbiome, where a diverse community of microorganisms interact with host tissues. These diseases, such as dental caries and periodontal infections, result from dysbiosis, the disruption of microbial balance (1). Pathogenic species such as *Streptococcus mutans* and *Porphyromonas gingivalis* can proliferate and form biofilms, which cause localized tissue damage. Disease susceptibility and progression are influenced by factors such as food, oral hygiene, systemic disease and host genetics (2). As the host attempts to regulate and alter microbial populations, immune responses play a critical role in determining the dynamics. Understanding the complexities of these relationships is critical for creating effective preventive and treatment interventions. The diagnosis of oral infectious diseases is critical for both individual oral health and public health management. Early intervention allows for the prevention of diseases, ultimately lowering the risk of complications and related systemic diseases (3). Oral infections are identified using a variety of diagnostic procedures. Clinical evaluation, which includes visual examination and probing, is still an important stage. Dental X-rays use radiographic imaging to visualize bone and tooth structure, which aid in establishing a provisional diagnosis. Microbiological examination entails collecting and culturing of oral samples in order to determine the pathogens responsible for the infection. Polymerase chain reaction (PCR) techniques enable molecular-level precision in detecting microbes, whereas next-generation sequencing (NGS) can provide a more comprehensive picture. Immunological methods, such as ELISAs, can also detect particular antibodies or antigens connected to oral infections. These diagnostic techniques enable doctors to develop treatment strategies and effectively follow disease development, ultimately improving oral health outcomes and general wellbeing.

Managing oral infectious diseases necessitates a multifaceted approach, incorporating preventive, medical, and surgical strategies. Prevention is the cornerstone, with strategies including oral hygiene prophylaxis and habit modifications (4). Effective plaque control through regular brushing and flossing mitigates the risk of dental caries and periodontal diseases by minimizing the growth of pathogenic biofilms (5). Medical management primarily involves antimicrobial therapy. Topical antimicrobial agents like chlorhexidine mouthwash is often prescribed for localized infections. Surgical intervention becomes necessary in advanced cases or when conservative methods prove insufficient. A comprehensive approach, tailored to the specific oral infection, is essential for effective management, preserving oral health, and preventing progression.

Some notable articles published in this issue are as follows: Morozova et al. emphasized the increased prevalence of dental diseases in children with cerebral palsy, which is caused by variables such as decreased salivation, altered pH, increase in salivary osmolarity, and changes in enzyme activity/sialic acid concentration, predisposing bacterial agglutination, biofilm and tooth plaque development.

Aimed to examine the association between patient-reported oral health outcomes and the dental service sector, a study by Song et al. uses a biopsychological model to analyze the correlation between reported oral health outcomes and the dental service sector, particularly the significance of trust in dentists. Oral health outcomes reported by patients were related to demographics, dental service sectors, and dentist trust. Lower trust in private dental care proved to be detrimental to oral health, thus underscoring the need for addressing inter-sector inequities and increasing dentist confidence, which are critical for improved oral health outcomes, especially among people who have poor trust in private dental services.

A systemic review performed by Uzochukwu et al. establishes the etiology behind Noma, of which microbial dysbiosis has proven to be pivotal in the pathogenesis of the disease. The review highlighted limitations and biases in the research designs, calling for further longitudinal studies which can provide stronger evidence for these findings.

A double-blinded study was carried out by Paszynska et al. to analyze the caries preventing effect of hydroxyapatite-toothpaste in adults. The primary result of the study was the percentage of patients who had no rise in the Decayed Missing Filled Surfaces (DMFS) index, which is a measure of caries. The trial was intended as a non-inferiority study, with the goal of demonstrating that the effect of hydroxyapatite toothpaste was not worse than the effect of fluoride toothpaste by more than a set margin of non-inferiority (20%). In terms of the primary outcome, hydroxyapatite toothpaste was found to be statistically non-inferior to fluoride toothpaste. This research shows that hydroxyapatite can be an effective and safe therapy for preventing dental cavities.

Qin et al. performed a study which investigated trends in edentulism rates in Chinese men and women from 1990 to 2019. Using data from the global burden of disease study 2019, the researchers discovered that while the crude incidence, prevalence, and years lived with disability (YLD) of edentulism increased year after year, age-standardized rates fell. The temporal effect demonstrated a progressive rise as a result of changing lifestyle. Cohort analysis revealed a declining trend, with earlier cohorts displaying a larger probability of tooth loss. Despite lowering standardized rates, edentulism continues to be a burden due to an aging population and an increasing period effect. Effective oral disease prevention methods, particularly among older women, are critical to reducing this burden.

The Research Topic highlights epidemiology, microbial dynamics, therapeutic methods, and preventive techniques in the confluence of Public Health Dentistry and oral disease diagnosis and management. The collaboration of oral infectious disease research efforts adds to a more thorough understanding of etiological variables, preventive measure outcomes and intervention efficacy. This synergy promotes the use of evidence-based practices, with an emphasis on early diagnosis, targeted medicines, and holistic health education, ultimately improving population-level oral health outcomes and lowering the societal burden of oral diseases.

This Research Topic compiles a series of original research studies and reviews combining scientific exploration and educational health initiatives, the goal is to heighten awareness about these diseases, institute preventive measures, and implement efficacious management protocols.

## Author contributions

DA: Conceptualization, Data curation, Formal analysis, Writing—original draft. SS: Conceptualization, Formal analysis, Supervision, Writing—review and editing. HY: Formal analysis, Writing—original draft. SP: Formal analysis, Project administration, Supervision, Writing—review and editing.
